# Impact of COVID-19 on the Vector-Borne Disease Research and Applied Public Health Workforce in the United States

**DOI:** 10.4269/ajtmh.21-1340

**Published:** 2022-02-22

**Authors:** Caroline T. Weldon, Scott C. Weaver, Kathryn H. Jacobsen

**Affiliations:** ^1^ Western Gulf Center of Excellence for Vector-Borne Diseases and Institute for Human Infections and Immunity, University of Texas Medical Branch, Galveston, Texas;; ^2^ University of Richmond, Richmond, Virginia

## Abstract

The coronavirus pandemic has imposed extraordinary demands on the public and environmental health workforce, including those who work on vector-borne disease (VBD) prevention and control. In late 2021, we surveyed more than 100 applied public health professionals, academic researchers, and others working on VBDs in the United States. They reported that the supply chain disruptions and limited access to facilities that impeded laboratory work in the early months of the COVID-19 pandemic in 2020 have largely resolved. However, many public health personnel across job types and career stages are still working fewer hours on VBDs than they did before the pandemic. Many reported that they expect it to take several years for VBD specialists to fully reengage with clinicians and the public, reinvigorate their partnerships and professional networks, and recover from interruptions to work productivity and professional development. Despite these challenges, most applied and academic VBD workers remain enthusiastic about their work and eager to advance this important area of infectious disease research and practice.

The COVID-19 pandemic required many public health workers in the United States to move from roles such as health education and program evaluation into contact tracing and other pandemic response activities. Many workers who specialized in areas such as chronic disease, maternal and child health, substance abuse, environmental health, and injury prevention were reassigned to coronavirus tasks. ^1^ There were also major disruptions to medical and public health research productivity, especially among laboratory researchers. ^2^ These applied and academic public health workforce challenges have had a significant adverse impact on vector-borne disease (VBD) work in the United States over the past 2 years.

In October 2021, after our research protocol was approved by the University of Texas Medical Branch (UTMB) Institutional Review Board, we contacted state and county health department personnel, researchers, and others in the United States. Gulf states who we knew were active in VBD research, prevention, control, surveillance, and/or other roles. These persons were invited to complete an anonymous online questionnaire about the impact of the pandemic on their work. We asked those initial contacts to share the survey link with other colleagues whose work focused at least in part on VBDs. This snowball sampling technique yielded more than 100 completed surveys from a diversity of participants by the end of November 2021. Slightly more than half of the respondents worked for state or county health departments or in other applied public health roles, and the remainder worked in academia. The states with the greatest number of respondents included Texas, Louisiana, Oklahoma, Arkansas, and Mississippi, but 20 states were represented. Despite the relatively small number of participants generated by this convenience sample, some clear trends emerged from this pilot study.

First, during the early months of the pandemic, many VBD workers experienced significant disruptions to acquisition of biological specimens and laboratory supplies as well as access to facilities, equipment, and other tools routinely used for VBD research, diagnosis, and surveillance. The volume of specimens being tested for VBDs decreased as fewer patients sought testing for any illnesses that were not severe and were not perceived to be related to COVID-19, and fewer healthcare providers ordered tests for diseases that were not suspected to be related to COVID-19. Global supply shortages further impaired the ability to conduct diagnostic tests and laboratory-based research. These barriers to VBD work have mostly resolved, but some persisted for nearly a year.

Second, most of the VBD workforce shifted to COVID-19 work in 2020. Most personnel in public health agencies had 100% of their time reassigned to contact tracing and other pandemic response roles in early 2020, and many of these individuals are still working fewer hours on VBDs than they did before the pandemic began. Many VBD researchers decreased the hours they were investing in VBD work so that they could add COVID research to their portfolios. Although some VBD workers and researchers had significantly increased total work hours, many had their paid work hours reduced. Reductions in the hours invested in VBD work were reported by both early career and established personnel. Almost everyone involved in applied VBD work or VBD research expected to be working at least part-time on VBDs 5 years from now, but their ability to continue working in the field was expected to be at least somewhat dependent on funding and prioritization of VBDs in the coming years.

Third, there were concerns that VBDs have been underdiagnosed and underreported in both 2020 and 2021. Without data on the true incidence of VBDs, it may have been difficult to secure the funding and other resources necessary to detect and respond to endemic and emerging VBD threats. Healthcare providers who were treating patients with COVID, the aftereffects of COVID, and chronic conditions that have worsened because of delayed care during the pandemic may have been underprepared to consider VBDs are part of differential diagnoses. Public awareness of VBDs likely decreased because of public health departments canceling educational events about VBD prevention and control and otherwise reducing contact with the public since the start of the pandemic. Outreach to clinicians, communities, and other audiences will be necessary to regain knowledge and resume good practices.

Fourth, it will take the VBD workforce several years to recover from work interruptions and lost professional development opportunities. Public health professionals reported having limited access to conferences, workshops, and other educational activities during the pandemic, even to online versions, due in many cases to reassigned duties. Many expressed concerns about how to make up for missed opportunities for in-person networking and hands-on learning. Academic researchers reported that they had generated more manuscripts than typical during the first year of the pandemic because they had time to write but were unable to collect new data. However, data collection gaps in 2020 and 2021 may mean fewer publications than typical for the next several years. Both applied and academic VBD personnel are eager to return to in-person training and conferences as soon as it is safe to gather.

A strong public and environmental health workforce is critical for preparing for the next pandemic and responding to existing threats, ^3^ including the increasing threat from VBDs. ^4^ The COVID-19 pandemic placed an unprecedented burden on national, state, county, and city health departments, adversely affecting the ability to recruit, train, and retain skilled public health professionals. ^5^ The pandemic also wreaked havoc on universities, and many scientists will need several years to recover from forced slowdowns or hiatuses in their productivity. ^6^ It will take even longer to secure adequate investment in VBD prevention and control, rebuild VBD partnerships, and ensure that VBD professionals have access to mentorship and networking. Despite these challenges, applied and academic VBD professionals remain enthusiastic about their work and are eager to grow this important area of infectious disease research and practice.
